# What is the role of puberty in the development of islet autoimmunity and progression to type 1 diabetes?

**DOI:** 10.1007/s10654-023-01002-7

**Published:** 2023-04-20

**Authors:** Essi J. Peltonen, Riitta Veijola, Jorma Ilonen, Mikael Knip, Harri Niinikoski, Jorma Toppari, Helena E. Virtanen, Suvi M. Virtanen, Jaakko Peltonen, Jaakko Nevalainen

**Affiliations:** 1 Unit of Health Sciences, Faculty of Social Sciences, Tampere University, Tampere, Finland; 2 Department of Pediatrics, Research Unit of Clinical Medicine, Medical Research Center, University of Oulu, Oulu, Finland; 3 Department of Children and Adolescents, Oulu University Hospital, Oulu, Finland; 4 Immunogenetics Laboratory, Institute of Biomedicine, University of Turku, Turku, Finland; 5 Pediatric Research Center, New Children’s Hospital, Helsinki University Hospital, Helsinki, Finland; 6 Research Program for Clinical and Molecular Metabolism, Faculty of Medicine, University of Helsinki, Helsinki, Finland; 7 Center for Child Health Research, Tampere University and Tampere University Hospital, Tampere, Finland; 8 Research Centre for Integrative Physiology and Pharmacology, Institute of Biomedicine, University of Turku, Turku, Finland; 9 Department of Pediatrics, Turku University Hospital, Turku, Finland; 10 Research Centre of Applied and Preventive Cardiovascular Medicine, University of Turku, Turku, Finland; 11 Centre for Population Health Research, University of Turku and Turku University Hospital, Turku, Finland; 12 Tays Research, Development and Innovation Center, Tampere University Hospital, Tampere, Finland; 13 Health and Well-Being Promotion Unit, Department of Public Health and Welfare, Finnish Institute for Health and Welfare, Helsinki, Finland; 14 Faculty of Information Technology and Communication Sciences, Tampere University, Tampere, Finland

**Keywords:** Puberty, Type 1 diabetes, Islet autoimmunity, Cohort study, Multi-state model

## Abstract

In many populations, the peak period of incidence of type 1 diabetes (T1D) has been observed to be around 10–14 years of age, coinciding with puberty, but direct evidence of the role of puberty in the development of T1D is limited. We therefore aimed to investigate whether puberty and the timing of its onset are associated with the development of islet autoimmunity (IA) and subsequent progression to T1D. A Finnish population-based cohort of children with *HLA-DQB1*-conferred susceptibility to T1D was followed from 7 years of age until 15 years of age or until a diagnosis of T1D (n = 6920). T1D-associated autoantibodies and growth were measured at 3- to 12-month intervals, and pubertal onset timing was assessed based on growth. The analyses used a three-state survival model. IA was defined as being either positive for islet cell antibodies plus at least one biochemical autoantibody (ICA + 1) or as being repeatedly positive for at least one biochemical autoantibody (BC1). Depending on the IA definition, either 303 (4.4%, ICA + 1) or 435 (6.3%, BC1) children tested positive for IA by the age of 7 years, and 211 (3.2%, ICA + 1)) or 198 (5.3%, BC1) developed IA during follow-up. A total of 172 (2.5%) individuals developed T1D during follow-up, of whom 169 were positive for IA prior to the clinical diagnosis. Puberty was associated with an increase in the risk of progression to T1D, but only from ICA + 1-defined IA (hazard ratio 1.57; 95% confidence interval 1.14, 2.16), and the timing of pubertal onset did not affect the association. No association between puberty and the risk of IA was detected. In conclusion, puberty may affect the risk of progression but is not a risk factor for IA.

**Supplementary Information:**

The online version contains supplementary material available at 10.1007/s10654-023-01002-7.

## Introduction

Type 1 diabetes (T1D) is an immune-mediated chronic disease in which insulin-producing β cells perish. The clinical disease is preceded by a preclinical phase of islet autoimmunity (IA) in which T1D-associated autoantibodies can be detected in the blood. [1] The majority of children diagnosed with T1D test positive for multiple autoantibodies during the preclinical phase, and the risk of subsequent progression to the clinical disease increases with increasing number of detected autoantibodies [2].

Over the last few decades, the incidence of T1D in children aged < 15 years has increased in many countries, although in more recent years, the incidence rates in some high-risk European countries, including Finland, have plateaued or even decreased [3, 4]. However, Finland still has the highest incidence globally with a rate of at least 52.2 per 100,000 person-years during 2015–2018 [4]. While genetics plays an important role in the development of the disease, the remarkable differences in incidence rates within countries and between neighboring countries with similar genetic backgrounds support the importance of environmental factors in the disease process [1].

Puberty and/or insulin resistance have been suggested as potential factors that may play a role in the development of T1D [5]. The incidence of IA peaks around 1–2 years, although it can occur at any age [6, 7], and a second peak of IA around puberty has been implicated, but the evidence is inconsistent [8, 9]. Nevertheless, the incidence of T1D has been observed to reach a peak around 10–14 years, coinciding with the pubertal period in many, although not all, populations and time periods [10–12].

Insulin sensitivity decreases at the onset of puberty, is at its lowest around mid-puberty, and recovers to pre-pubertal levels once puberty has fully passed [13, 14]. This decrease in insulin sensitivity may contribute to the progression of T1D, especially in people already at high risk of developing the disease [15–19], but there are indications that the role of insulin resistance as an accelerator of the disease process is minor [20]. While etiology of IA and T1D remains elusive, endoplasmic reticulum (ER) stress has been proposed to play an important role in beta cell dysfunction that might lead to antigen release and triggering of the autoimmune response in susceptible individuals [21]. Rapid pubertal growth and puberty-related insulin resistance increase the insulin demand and cause thereby ER stress in the beta cells rendering them vulnerable. Similarly, over-nutrition, obesity and obesity-induced insulin resistance challenge the beta cells, overwhelming their capacity to properly handle insulin production. Beta cells use an unfolded protein response to increase insulin production during high metabolic demand, and failure of this may lead to antigen release [21]. However, the direct evidence of the role of puberty in the development of T1D, including intermediate IA, is limited. We therefore aimed to study whether puberty and the age at onset of it are associated with the development of IA and subsequent progression from IA to T1D in the large, population-based Finnish Type 1 Diabetes Prediction and Prevention (DIPP) cohort of young children with *HLA-DQB1* -conferred susceptibility to T1D, who were followed up intensively from birth through puberty.

## Material and methods

### Participants

The data originated from the Finnish prospective population-based DIPP Study [22]. Participants in the DIPP birth cohort were born at the Turku (1994–), Oulu (1995–), and Tampere (1997–) University Hospitals. Newborn infants were screened for *HLA-*conferred genetic susceptibility to T1D using cord blood samples. *HLA* class II genes are the strongest contributors to the genetic disease susceptibility, being responsible for approximately 50% of the heritability [23]. In addition, also other genes with more than 70 identified contribute to T1D development [24].

Originally, children carrying the genotypes HLA-DQB1*02/*03:02 and HLA-DQB1*03:02/x (x ≠ *02, *03:01, or *06:02/3 until March 1997, and x ≠ *02, *03:01, or *06:02 thereafter) were selected for follow-up [22]. Later on, more *DQB1* and *DQA1* alleles and subtypes of DRB1*04 alleles were included in the screening to increase the specificity and sensitivity of the procedure [25]. Siblings of the participants were also tested for *HLA-*conferred susceptibility and invited for follow-up if an increased risk was detected.

**Fig. 1:**
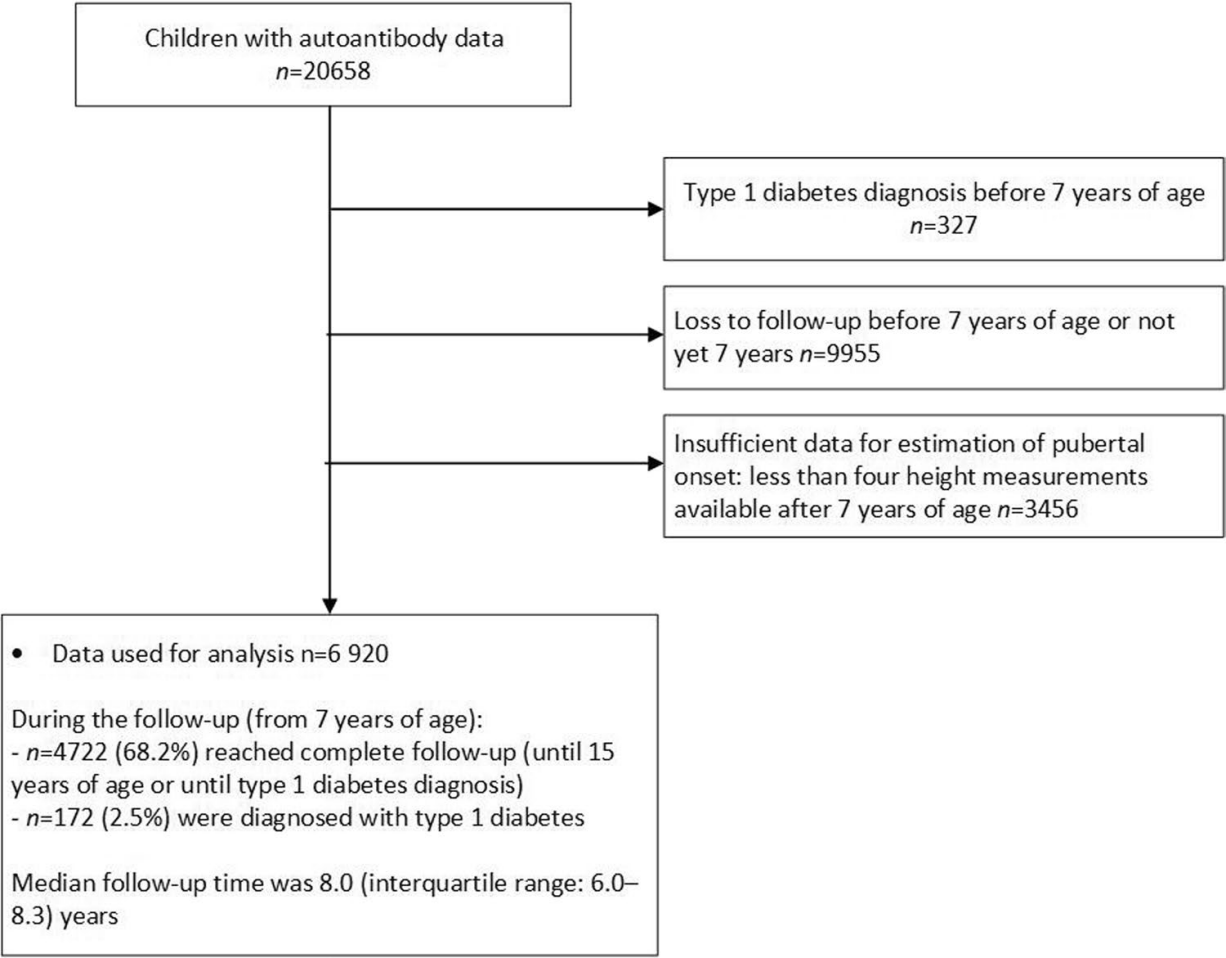
Flow chart of the compilation of the study population for analysis

The original data included all DIPP participants born before 2020 and their genetically susceptible siblings who participated in the follow-up. Data from 7 years of age were used in the analyses, and participants who had at least four height measurements were included in the assessment of pubertal onset (n_girls_ = 3118, n_boys_ = 3803). Of these, all who had not developed T1D before 7 years of age were included in the final analyses (n = 6920, Fig. 1). Children who tested positive for IA by 7 years of age contributed only to the analysis of the progression hazards.

### Background characteristics

Information on sex was collected as part of a structured questionnaire completed in the hospital after delivery. The child’s body mass index (BMI) at different ages was calculated using the available height and weight measurements obtained during follow-up. To assess BMI at 7 years of age, a polynomial spline mixed-effects model with random intercept and random slopes was fitted to BMI values by age, and each child’s BMI at 7 years was obtained from the individual BMI–age trajectory. For the analyses, the participants were classified into two categories based on the derived BMI value as follows: overweight (ISO-BMI > 25, including overweight and obese) and other (including normal weight and underweight) children, based on the Finnish ISO-BMI classification for children [26]. The ISO-BMI classification defines age-specific BMI cut-off points for underweight, overweight, and obesity for ages 2 to 18 years among Finnish children.

### Follow-up characteristics

Participants were monitored for T1D-associated autoantibodies, growth, and weight up to the age of 15 years or until diagnosed with T1D. If autoantibodies were detected before the age of 15 years but the clinical disease was not diagnosed by that point, monitoring would continue. For children born until 2010, antibodies were analyzed at the ages of 3, 6, 12, 18, and 24 months and then annually at the Tampere and Oulu centers and at 3-month intervals until the age of 2 years and then semiannually at the Turku center. For children born since 2010, antibodies were analyzed at the ages of 3, 6, 9, 12, and 24 months and then annually at all centers. After the possible detection of autoantibodies, the participants were followed up at 3-month intervals. Data for T1D diagnosis were obtained from the Finnish Pediatric Diabetes Registry [4] or from the study clinics.

### Immunological methods

Four T1D -associated autoantibodies were analyzed, with islet cell antibodies (ICA) used as the primary screening tool for children born before the end of 2002. If a child tested positive for ICA, all the child’s preceding (starting from birth) and subsequent samples were analyzed for three biochemical autoantibodies: insulin autoantibodies (IAA), glutamic acid decarboxylase antibodies (GADA), and islet antigen 2 antibodies (IA-2A). For children born from 2003 onwards, all four autoantibodies from all their samples were regularly analyzed. ICAs were quantified using a standard indirect immunofluorescence method and IAAs, GADAs, and IA-2As using specific radio-binding assays, as described previously [27]. The cut-off for autoantibody positivity was 2.5 JDFU (Juvenile Diabetes Foundation units) for ICA, 3.48 relative units (RU) for IAA, 5.36 RU for GADA and 0.43 RU for IA-2A. Transplacentally transferred autoantibodies were monitored from the maternal samples and from the children’s early samples to exclude those from the analyses.

### Definition of outcomes

IA and clinical T1D diagnosis observed after the age of 7 years were used as the outcomes of the study. Because IA is not an unambiguous state, we used two different definitions: 1) repeated positivity for ICA together with at least one biochemical autoantibody (ICA + 1), and 2) repeated positivity for at least one biochemical autoantibody (BC1). As the risk of progression increases significantly with increasing numbers of detected autoantibodies [2], BC1 can be considered a less stringent definition for IA than ICA + 1.

### Determination of age at pubertal onset

Age at pubertal onset was assessed based on individual growth characteristics—peak height velocity (cm per year) and age at peak height velocity (years)—and on overweight status (based on ISO-BMI [26]) before the pubertal growth spurt. The assessment was carried out using a previously constructed and validated time-to-pubertal onset model [28]. The children were also divided (at the 33rd and 67th percentiles) into three equally sized groups based on age at pubertal onset; the age cut-off points for the groups were 10.0 and 11.0 years for girls and 11.3 and 11.8 years for boys.

### Ethics

Parents gave their written informed consent to genetic testing of their newborn infant and for participation in the follow-up. The study adheres to the Declaration of Helsinki, and the ethics committee of Northern Ostrobothnia Hospital District approved the study protocol. Data were pseudonymized before the corresponding author accessed them for analysis.

### Statistical analysis

#### The three-state survival model

Progressive three-state survival models [29] for continuous time data were used to study the association between puberty and the development of IA and progression to T1D. The hazards were assumed to follow a Weibull distribution. We defined state 1 as being negative for IA without T1D, state 2 as having IA without T1D, and state 3 as being diagnosed with clinical T1D. Time to IA was considered to be interval censored between the last negative IA measurement and the first positive IA measurement, meaning that the true transition time was unknown, although it was known to lie within a specific time interval [30]. T1D was the absorbing state—backward transition from this state was not considered possible, and entry into this state ended the follow-up. Three irreversible transitions were therefore possible: 1 → 2 (IA), 1 → 3 (direct transition to T1D), and 2 → 3 (progression). A few 1 → 3 transitions were considered to be unobserved 1 → 2 → 3 paths based on the rarity of the T1D diagnosis without detected autoantibodies [6]. This three-state model is illustrated in Supplementary Fig. 1.

In addition to the observed transitions, the possibility of unobserved transitions was accounted for in the models. Unobserved transitions for IA represented children under follow-up who had no measurements to assess their status at 7 years (potential IA at 7 years) followed by an observed IA (ICA + 1: n = 16; BC1: n = 27) together with those with a T1D diagnosis but without initial observation of IA (n = 3). The latter were also considered to be unobserved transitions from IA to T1D (progression).

The models were adjusted for the sex of the child (girl or boy) and overweight status (underweight/normal weight or overweight/obese) at 7 years. The potential modifying effect of the actual timing of pubertal onset (divided into three categories by tertiles) on the association between puberty and the transition-specific risks was investigated by adding the corresponding interaction term to the model. Additional analyses for a modifying effect of the pubertal timing were implemented with cut-off points for early and late puberty defined by the age 9 and 12 years for girls, and 10 and 13 years for boys, respectively. Type I errors due to multiple testing were controlled for using the false discovery rate (FDR) method [31] for the adjusted results (four family-wise comparisons were accounted for). The FDR was set at 0.05, and a result was considered a discovery if the p-value was smaller than the corresponding FDR critical value.

#### The pubertal effect function

Puberty was added to the three-state model as a novel time-dependent function. Different origins, durations, and shapes for the effect were considered as alternative forms of pubertal influence. In the function definition, *origin* was defined as the initiation of the effect—whether the pubertal effect starts at the estimated onset or before it; *duration* was defined as the time period of the pubertal effect; and the different *shapes* of the pubertal effect consisted of ramping-up, steady, and fading-off periods, each accounting for different proportions of the overall duration of the effect but with equal durations for ramping-up and fading-off. The functional form of the pubertal effect is illustrated in detail in the Supplementary Methods.

We considered a range of alternative functional forms. The origin was assumed to occur at onset of puberty or 1 year before; the duration ranged from 1 to 4 years, at 1-year intervals; and the shape was expressed as a percentage of the entire duration at 10% intervals (i.e., 11 options), with ramping-up and fading-off periods being equally long. This led to close to one hundred presumed possible forms of the pubertal effect. It was assumed that the same origin, duration, and shape applied to both transitions in each model, but their regression coefficients could be different.

The pubertal effect function used was chosen in a data-driven manner based on the Akaike information criterion (AIC) by choosing the smallest average AIC over the different IA definitions. The final multi-state models included a pubertal effect with an origin at 1 year before the estimated onset of puberty, a duration of 3 years, and a shape comprising a 0.3-year (10%) ramping-up, a 2.4-year (80%) steady, and a 0.3-year (10%) fading-off period. We verified the performance of the data-driven model selection by implementing a small simulation study, which demonstrated probability of approximately 80% for detecting a pubertal effect as well as the ability to correctly estimate the relative risk even under a slight misspecification of the functional form (see Supplementary Methods).

#### Sensitivity analysis

We investigated the sensitivity of the results in several ways. The possibility of an error in the growth-based estimation of pubertal onset was evaluated by fitting the same model to ten datasets, with the timing of onset randomly drawn from a normal distribution corresponding to the individual’s pubertal onset prediction interval obtained from the time-to-pubertal onset model [28]. Results from the models were combined using Rubin’s Rules [32].

To verify that the significant findings were not specific to the choice of a particular functional form, we tested a range of models with different origins, durations, and/or shapes and compared them to the results using the chosen functional form.

Finally, additional analyses defining IA as repeated positivity for at least two biochemical autoantibodies (BC2) were performed to determine whether the definition of IA affected the results. In addition, a Cox regression model on progression to T1D was fitted as a sensitivity analysis. The analysis included individuals being positive for IA and the time origin was at 7 years. In addition to the adjustment for the confounding factors, the age at IA was adjusted for because early seroconversion to islet autoantibodies is known to be associated with a faster rate of progression to T1D [6]. Puberty was used as a time dependent covariate lasting from 1 year before onset until 2 years after.

## Results

Of the 6920 participants, 3117 (45.0%) were girls and 1572 (22.7%) were overweight or obese at the age of 7 years. The mean estimated age at pubertal onset was 10.6 (sd: 0.98) years for girls and 11.6 (sd: 0.62) years for boys.

Depending on the IA definition, either 303 (4.4%, ICA + 1) or 435 (6.3%, BC1) children tested positive for IA by the age of 7 years and during the follow-up, 211 (3.2%, ICA + 1) or 198 (5.3%, BC1) developed IA. There were 16 (0.2%, ICA + 1) or 27 (0.4%, BC1) children who had no measurements to assess IA-status at the age of 7 years but were observed to be positive for IA during the follow-up (Table 1). Among all the participants, 172 (2.5%) developed T1D during the follow-up, at a median age of 11.3 (interquartile range: 9.3–13.3) years, of whom 169 tested previously positive for IA (Table 1). Neither the sex of the child nor overweight status at 7 years of age were found to be associated with the risk of IA or the risk of progression to T1D (Table 2).

**Table 1:** Distribution of type 1 diabetes development status at 7 years of age (the beginning of follow-up) for different outcomes, and the numbers and proportions of observed transitions during follow-up

	Status at 7 years of age, n (% of N = 6920)				Number of transitions^a^	
	No IA	IA	Potential IA^b^	Potential IA^b^ followed by IA	IA^c^	Progression^d^
ICA + 1	6599 (95.4%)	303 (4.4%)	2 (0.03%)	16 (0.2%)	211 (3.2%)	169 (31.9%)
BC1	3724 (53.8%)	435 (6.3%)	2734 (39.5%)	27 (0.4%)	198 (5.3%)	169 (25.6%)

IA, islet autoimmunity; T1D, type 1 diabetes; Progression, progression from IA to T1D

^a^In addition to observed transitions, the possibility of unobserved transitions was accounted for in the model

^b^Children being followed up (T1D-free) who had no measurements to assess IA status at 7 years of age

^c^Observed transitions with the proportions of children observed to be IA-negative at 7 years of age

^d^Observed transitions with the proportions of children observed to be IA-positive during follow-up (at 7 years of age or with transition during follow-up)

**Table 2:** Distribution of background characteristics and their association with the risk of islet autoimmunity (IA) and with the risk of progression to type 1 diabetes (T1D) for different outcomes; numbers include both unobserved and observed transitions

	Transition	Sex					Overweight status at 7y				
		Girl^a^ n = 3117		Boy n = 3803		HR (95% CI)	Underweight/normal weight^a^ n = 5348		Overweight/obese n = 1572		HR (95% CI)
		n (%)	Total N	n (%)	Total N		n (%)	Total N	n (%)	Total N	
ICA + 1	IA^b^	92 (3.1)	2994	138 (3.8)	3623	1.26 (0.95, 1.67)	169 (3.3)	5102	61 (4.0)	1515	1.18 (0.87, 1.61)
	Progression^c^	76 (35.3)	215	96 (30.2)	318	0.80 (0.59, 1.09)	142 (34.2)	415	30 (25.4)	118	0.82 (0.56, 1.22)
BC1	IA^b^	92 (3.1)	2935	136 (3.8)	3550	1.29 (0.97, 1.71)	172 (3.4)	5002	56 (3.8)	1483	1.10 (0.80, 1.52)
	Progression^c^	76 (27.7)	274	96 (24.7)	389	0.99 (0.73, 1.35)	142 (27.4)	518	30 (20.7)	145	0.74 (0.49, 1.10)

IA, islet autoimmunity; Progression, progression from IA to type 1 diabetes; HR, hazard ratio; CI, confidence interval

^a^Reference group

^b^Total N: those not observed to be IA-positive at 7 years of age; n: those who developed IA or were diagnosed with T1D with unobserved IA

^c^Total N: those observed to be IA-positive during follow-up (at 7 years of age or with transition during follow-up) or diagnosed with T1D with unobserved IA; n: those diagnosed with T1D

**Fig. 2:**
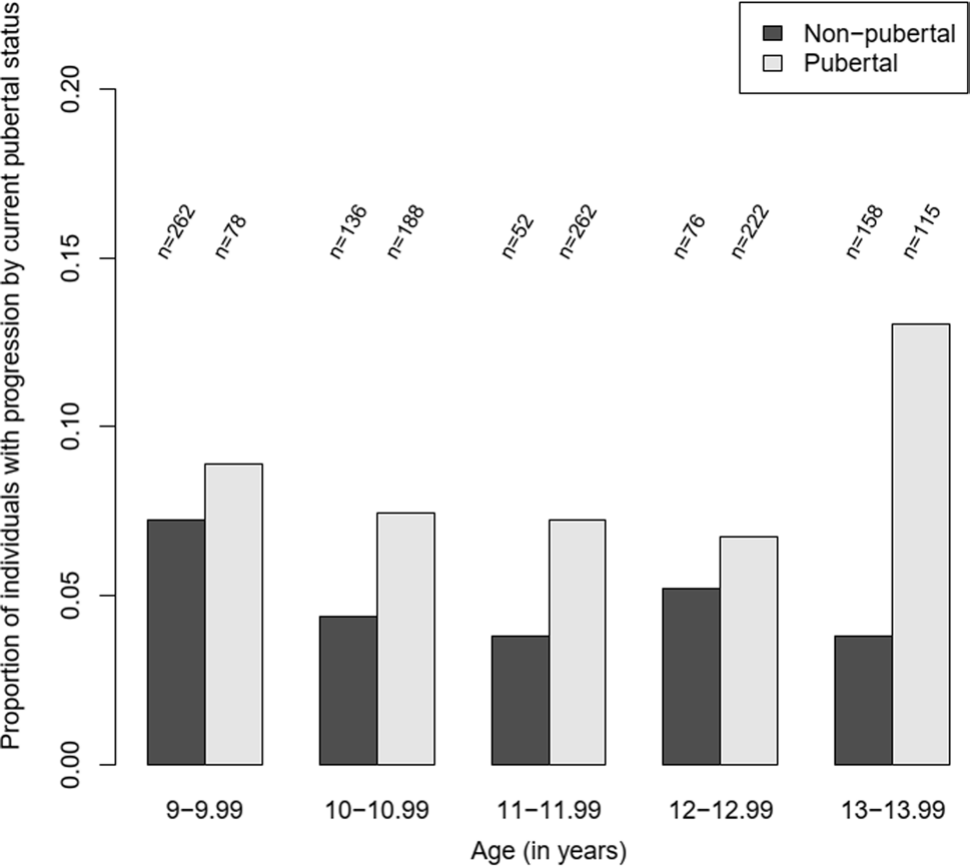
Proportions of individuals with progression from ICA + 1-defined islet autoimmunity to type 1 diabetes within every one-year interval. The numbers (n) of non-pubertal and pubertal individuals who were positive for islet autoimmunity and still in the follow-up are presented above the bars. Pubertal status was defined based on the pubertal period, which was considered to be from 1 year before estimated onset to 2 years after, consistent with the model. Only time intervals with more than one pubertal progression are presented

Despite trending in the direction of elevated risk, puberty was not significantly associated with the risk of IA. However, puberty was associated with the risk of progression from ICA + 1-defined IA to T1D and the hazard for a pubertal child was 1.57 (95% CI 1.14, 2.16) times that of a non-pubertal child (Table 3, Supplementary Fig. 2). Figure 2 illustrates the proportions of non-pubertal and pubertal individuals with progression from ICA + 1-defined IA to T1D within one-year intervals. No association between puberty and the risk of progression was detected with the BC1 definition for IA.

**Table 3:** Number of transitions for different outcomes with hazard ratios for puberty, both overall and by timing of pubertal onset; numbers include both unobserved and observed transitions

	Transition	Timing of pubertal onset	n (%)^a^	Unadjusted model HR (95% CI)	*P*-value^b^	Adjusted model^c^ HR (95% CI)
ICA + 1	IA	Before 1st tertile	80 (3.7)	1.33 (0.87, 2.03)	0.542	
		1st–2nd tertile	87 (3.7)	1.48 (0.94, 2.32)		
		After 3rd tertile	63 (3.0)	0.88 (0.49, 1.57)		
		Overall	230 (3.5)	1.30 (0.94, 1.81)		1.25 (0.90, 1.74)
	Progression	Before 1st tertile	47 (28.0)	1.41 (0.88, 2.26)	0.659	
		1st–2nd tertile	83 (37.2)	1.53 (0.95, 2.47)		
		After 3rd tertile	42 (29.6)	1.96 (1.24, 3.11)		
		Overall	172 (32.3)	1.63 (1.19, 2.24)		1.57 (1.14, 2.16)^d^
BC1	IA	Before 1st tertile	68 (3.2)	1.05 (0.64, 1.70)	0.296	
		1st–2nd tertile	98 (4.3)	1.72 (1.10, 2.68)		
		After 3rd tertile	62 (3.0)	0.76 (0.40, 1.47)		
		Overall	228 (3.5)	1.18 (0.83, 1.68)		1.24 (0.87, 1.77)
	Progression	Before 1st tertile	47 (22.4)	1.23 (0.77, 1.98)	0.954	
		1st–2nd tertile	83 (30.1)	1.28 (0.79, 2.08)		
		After 3rd tertile	42 (23.7)	1.37 (0.86, 2.19)		
		Overall	172 (25.9)	1.30 (0.95, 1.79)		1.31 (0.95, 1.80)

IA, islet autoimmunity; Progression, progression from IA to type 1 diabetes; HR, hazard ratio; CI, confidence interval

^a^Proportion of (for IA) those not observed to be IA-positive at 7 years of age or (for progression) those observed to be IA-positive during follow-up (at 7 years of age or with transition during follow-up) or those diagnosed with T1D with unobserved IA

^b^Wald test for equality of timing-specific HRs

^c^Adjusted for sex and overweight status at 7 years of age

^d^Statistically significant after correction for multiple testing, with p-value of 0.0056 and FDR critical value of 0.0063

The timing of pubertal onset did not affect the association between puberty and the risk of progression from IA to T1D in the main analysis (Table 3). In the additional analyses with categories of early, normal and late puberty, there was a weak indication of larger effect for early onset than later onset of puberty (Supplementary Table 2). However, the number of individuals with early puberty and positivity for IA was very low (n = 5).

All the results remained similar in the sensitivity analyses after considering uncertainty in the estimation of puberty onset (data not shown). The statistically significant finding of an association between puberty and ICA + 1-defined progression to T1D was consistent under different forms of pubertal effect functions, which did not markedly weaken the model fit (AIC; Supplementary Table 1). The results with the additional BC2 definition for IA were similar to the results using the BC1 definition (data not shown). The results of the Cox regression model for progression to T1D after IA were very similar to the main results (Supplementary Table 3).

## Discussion

We investigated the role of puberty in the development of IA and further progression to T1D. No association was found between puberty and the risk of IA, but puberty was associated with the risk of progression to T1D using the ICA + 1 definition for IA. The hazard ratio of progression to T1D from BC1-defined IA was in the same direction, but did not reach statistical significance. The timing of pubertal onset did not modify the association.

This study is among the first assessments of the role of puberty in T1D development. Insulin resistance is a normal feature of puberty, and previous studies have implied that it may contribute to the progression of T1D [15–19]. From that perspective, the potential association found between puberty and the higher risk of progression to T1D in the present study is consistent with the existing literature. In addition, the production of growth hormone and insulin-like growth factor-1 increases during puberty, and both have been linked to insulin resistance during puberty [33, 34], pointing to the possibility that they may contribute to the association found.

Based on a large Biobank study from the UK, there are associations between the timing of puberty as either early or late and higher risks of numerous adverse outcomes, including gynecological, cardio-metabolic, gastrointestinal, musculoskeletal, and neuro-cognitive, as well as a range of cancers in both men and women [35]. Since pubertal timing seems to have a broad influence on later health, we wanted to assess the effect of the timing on the association between puberty and the outcomes. Our analyses did not find any notable differences in the particular associations between the pubertal timing categories.

The results were somewhat sensitive to the specification of the pubertal effect function. The AIC suggested models in which an association between puberty and progression was present, and we demonstrated through sensitivity analyses that a misspecification of the effect could lead to a loss of significance, although not a change in the direction of the effect. This underlines the need to model this effect carefully. The identified duration for the pubertal effect closely agreed with the previously observed durations of puberty, which were 4.1 years for girls and 3.8 years for boys [36].

The major strength of the current study is the large and unique longitudinal cohort with its long follow-up for autoantibodies, growth, and T1D. The use of the multi-state model enabled a state representation that is well suited to the T1D disease process because autoantibodies usually precede the clinical disease. Modeling enabled the risk factors for progression to be studied while considering the child’s preceding states, and the flexible inclusion of the pubertal effect as a function for the multi-state model enabled a comparison of different potential forms of the effect.

Limitations of the study include the lack of systematic biochemical autoantibody (IAA, GADA, and IA-2A) measurements. Among children born before 2003, ICA was used as a primary screening tool, meaning that biochemical antibodies were detected only if ICA was positive. This may have affected the results regarding the biochemical endpoint because some autoantibody-positive measurements may have been missed. However, since a positive test for biochemical autoantibodies without the appearance of ICA is uncommon [6, 37], we believe that it is unlikely that this would have had a major effect on the results. In addition, the previous simulation study in the same data showed that the effect of selective sampling on the regression coefficients for the secondary autoantibody outcomes is minor [38]. Antigen specificity of ICA is not known, and it is of interest that sensitivity analysis with a BC2 definition gave similar results to those of BC1, suggesting possible contribution of other autoantibodies than IAA, GADA, or IA-2A in the ICA + BC1 group, e.g., zinc transporter 8 autoantibodies. Another limitation is that the study did not involve a physical pubertal follow-up, and the pubertal onset timings were estimated on the basis of a validated growth assessment method [28]. The effect of IA on later growth, including pubertal growth, is unknown, which may in turn influence the pubertal timing assessment. Hence, reverse causation is possible. As some of the children were excluded due to the study design and by requiring sufficiently many height measurements for pubertal timing assessment, immortal time bias cannot be ruled out. The generalizability of the results might also be limited because the current study was conducted in subjects with increased HLA-conferred genetic risk for T1D.

In conclusion, this study suggests that puberty might have an accelerating role in progression to T1D but has no role in the development of IA. The study is novel in its investigation of the direct role of puberty—not via insulin sensitivity—in the development of T1D, together with the ability to model disease progression and puberty together. Based on our results, the previously observed peak in T1D incidence during adolescence [1] could be associated with pubertal development.

## Supplementary Information

Below is the link to the electronic supplementary material.

Supplementary file1 (DOCX 56 kb)

Supplementary file2 (DOCX 156 kb)
