# Supplementary material for: What is the role of puberty in the development of islet autoimmunity and progression to type 1 diabetes?
Source: Eur J Epidemiol. 2023 Apr 20;38(6):689–97. doi: 10.1007/s10654-023-01002-7 (PMC10232567; doi:10.1007/s10654-023-01002-7)
Supplement: Supplementary file 1 — Supplementary file1 (DOCX 56 kb) [file 10654_2023_1002_MOESM1_ESM.docx]

**Supplementary Figures and Tables**

**What is the role of puberty in the development of islet autoimmunity and progression to type 1 diabetes?**

*European Journal of Epidemiology*

Essi J. Peltonen, Riitta Veijola, Jorma Ilonen, Mikael Knip, Harri Niinikoski, Jorma Toppari, Helena E. Virtanen, Suvi M. Virtanen, Jaakko Peltonen, Jaakko Nevalainen

**Correspondence:**

Essi J. Peltonen, Unit of Health Sciences, Faculty of Social Sciences, Tampere University, Tampere, Finland; E-mail: [essi.peltonen@tuni.fi](mailto:essi.peltonen@tuni.fi)


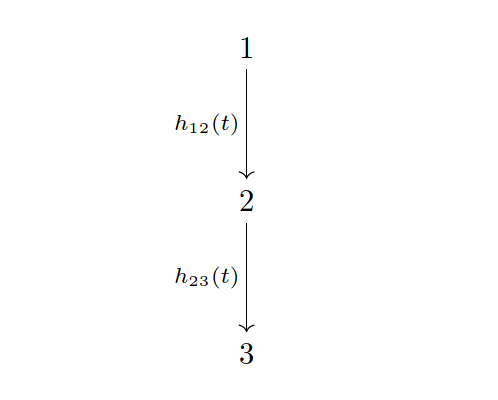


**Supplementary Fig. 1** The structure of the three-state survival model. State 1 corresponds to non-diabetic and islet autoimmunity negativity, state 2 to islet autoimmunity, and state 3 type 1 diabetes diagnosis. $h_{rr'}\left( t \right)$, with $r,r^{'}=1,2,3$ are the transition-specific hazards. They are of the form $h_{rr'}\left( t \right)=h_{rr'}^{0}\left( t \right)e^{\boldsymbol{\beta}_{rr'}^{'}\boldsymbol{X+}\gamma_{rr'}Z(t)}$, where $h_{rr'}^{0}\left( t \right)$ are the Weibull distributed transition-specific baseline hazards, $\boldsymbol{X}$ is a vector of baseline covariates including sex and overweight status at 7 years, with corresponding regression coefficients ${\boldsymbol{\beta}'}_{rr'}$ , and $Z(t)$ is a value of pubertal function at time *t*, with corresponding regression coefficients $\gamma_{rr'}$


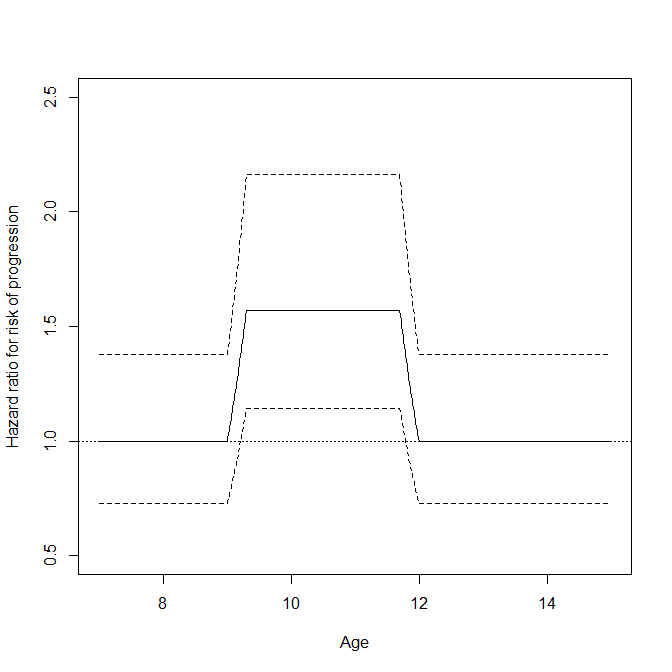


**Supplementary Fig. 2** The hazard ratio with 95% confidence intervals for progression from ICA+1 defined islet autoimmunity during the pubertal period compared to a non-pubertal period for the child with the estimated pubertal onset occurring at 10 years of age. Then according to the model, the origin of the pubertal effect is assumed to be at 9 years, duration 3 years, and shape includes a 0.3-year (10%) ramping up, 2.4-year (80%) flat and 0.3-year (10%) fading off periods

**Supplementary Table 1** Sensitivity analyses of different choices for the pubertal effect origin, shape, and/or duration on the results with ICA+1 defined islet autoimmunity and progression from islet autoimmunity to type 1 diabetes. The results presented are unadjusted

| **Components changed** | **Duration** | **Origin** | **Shape (% of duration)** | **Islet autoimmunity^a^** | **Progression^a^** | **AIC^b^** |
| --- | --- | --- | --- | --- | --- | --- |
| **None (the model used)** | 3 years | 1 year before onset | 10/80/10 | 1.31 (0.94, 1.81) | **1.63 (1.19, 2.24)** | 4547.5 |
| **Origin** | 3 years | At onset | flat | 1.20 (0.84, 1.73) | 1.38 (0.99, 1.92) | 4554.2 |
|  |  |  | 10/80/10 | 1.30 (0.90, 1.89) | **1.41 (1.01, 1.99)** | 4553.1 |
| **Shape** | 3 years | 1 year before onset | flat | 1.24 (0.90, 1.71) | **1.57 (1.16, 2.13)** | 4548.9 |
|  |  |  | 30/40/30 | 1.38 (0.95, 1.99) | **1.55 (1.08, 2.22)** | 4550.7 |
| **Duration** | 2 years | 1 year before onset | flat | 1.15 (0.82, 1.61) | 1.20 (0.85, 1.70) | 4557.4 |
|  |  |  | 10/80/10 | 1.14 (0.81, 1.62) | 1.20 (0.84, 1.73) | 4557.3 |
| **Origin + duration** |  | At onset | flat | 1.27 (0.88, 1.08) | **1.52 (1.08, 2.14)** | 4551.7 |
|  |  |  | 10/80/10 | 1.35 (0.93, 1.97) | **1.49 (1.04, 2.14)** | 4551.8 |

^a^ Numbers are hazard ratios with 95% confidence intervals in parentheses

^b^ Akaike information criterion

**Supplementary Table 2** Number of transitions for different outcomes with hazard ratios for puberty, both overall and by timing^b^ of pubertal onset; numbers include both unobserved and observed transitions

|  | **Transition** | **Timing of pubertal onset** | **n (%)^a^** | **Unadjusted model^b^**  **HR (95% CI)** | **P-value^c^** |
| --- | --- | --- | --- | --- | --- |
| **ICA+1** | **IA** | *Early* | 1 (1.1) | 0.92 (0.12, 6.75) | 0.901 |
|  |  | *Normal* | 219 (3.5) | 1.39 (0.99, 1.94) |  |
|  |  | *Late* | 10 (3.2) | 0.68 (0.13, 3.60) |  |
|  |  | *Overall* | 230 (3.5) | 1.30 (0.94, 1.81) |  |
|  | **Progression** | *Early* | 3 (60.0) | **5.72 (1.71, 19.10)** | **0.032** |
|  |  | *Normal* | 165 (32.5) | **1.56 (1.13, 2.15)** |  |
|  |  | *Late* | 4 (23.5) | 1.08 (0.26, 4.57) |  |
|  |  | *Overall* | 172 (32.3) | **1.63 (1.19, 2.24)** |  |
| **BC1** | **IA** | *Early* | 1 (1.1) | 0.81 (0.11, 6.04) | 0.668 |
|  |  | *Normal* | 216 (3.6) | 1.23 (0.87, 1.76) |  |
|  |  | *Late* | 62 (3.0) | 0.16 (0.01, 4.93) |  |
|  |  | *Overall* | 228 (3.5) | 1.18 (0.83, 1.68) |  |
|  | **Progression** | *Early* | 3 (60.0) | **6.03 (1.81, 20.04)** | **0.007** |
|  |  | *Normal* | 165 (26.0) | 1.27 (0.92, 1.76) |  |
|  |  | *Late* | 4 (20.0) | 0.82 (0.19, 3.46) |  |
|  |  | *Overall* | 172 (25.9) | 1.30 (0.95, 1.79) |  |

IA: islet autoimmunity; Progression: progression from IA to type 1 diabetes; HR: hazard ratio; CI: confidence interval

^a^ Proportion of (for IA) those not observed to be IA-positive at 7 years of age or (for progression) those observed to be IA-positive during follow-up (at 7 years of age or with transition during follow-up) or those diagnosed with T1D with unobserved IA.

^b^ The age cutoff points for the pubertal timing categories were 9 and 12 years for girls, and 10 and 13 years for boys.

^c^ Wald test for equality of timing-specific HRs.

**Supplementary Table 3** Risk of progression to type 1 diabetes with puberty and confounding factors based on the Cox regression model for individuals tested positive for islet autoimmunity (n=530)

|  | **Unadjusted model^a^** |  | **Adjusted model^b^** | | |  |
| --- | --- | --- | --- | --- | --- | --- |
| **Variable** | **HR (95% CI)** |  | **Total n (%)** | **T1D n (%)^d^** | **HR (95% CI)** | |
| **Puberty**^c^ | 1.60 (1.03, 2.48) |  | - | - | 1.56 (1.02, 2.41) | |
| **Age at IA (in years)** | 0.89 (0.85, 0.92) |  | - | - | 0.89 (0.85, 0.92) | |
| **Sex** |  |  |  |  |  | |
| Girl |  |  | 214 (40.4) | 75 (10.1) | 1^e^ | |
| Boy |  |  | 316 (59.6) | 94 (8.8) | 0.78 (0.58, 1.06) | |
| **Overweight status** |  |  |  |  |  | |
| Underweight/normal weight |  |  | 412 (77.7) | 139 (10.1) | 1^e^ | |
| Overweight/obese |  |  | 118 (22.3) | 30 (7.0) | 0.81 (0.55, 1.21) | |

IA: islet autoimmunity; HR: hazard ratio; CI: confidence interval; T1D: type 1 diabetes

^a^ Unadjusted model was still adjusted for the age at IA

^b^ Adjusted for the age at IA, sex and overweight status at 7 years of age

^c^ Time-dependent puberty indicator (binary variable) with origin of puberty being at 1 year before the estimated onset and duration 3 years

^d^ Number and proportion of individuals with progression from IA to T1D (totally n=169)

^e^ Reference group
