# Supplementary material for: What is the role of puberty in the development of islet autoimmunity and progression to type 1 diabetes?
Source: Eur J Epidemiol. 2023 Apr 20;38(6):689–97. doi: 10.1007/s10654-023-01002-7 (PMC10232567; doi:10.1007/s10654-023-01002-7)
Supplement: Supplementary file 2 — Supplementary file2 (DOCX 156 kb) [file 10654_2023_1002_MOESM2_ESM.docx]

**Supplementary Methods**

**What is the role of puberty in the development of islet autoimmunity and progression to type 1 diabetes?**

*European Journal of Epidemiology*

Essi J. Peltonen, Riitta Veijola, Jorma Ilonen, Mikael Knip, Harri Niinikoski, Jorma Toppari, Helena E. Virtanen, Suvi M. Virtanen, Jaakko Peltonen, Jaakko Nevalainen

**Correspondence:**

Essi J. Peltonen, Unit of Health Sciences, Faculty of Social Sciences, Tampere University, Tampere, Finland; E-mail: [essi.peltonen@tuni.fi](mailto:essi.peltonen@tuni.fi)

1. **Definition of the three-state survival model**

We modelled the transition-specific hazard from state $r$ to state $r’$ $\left( r,r^{'}=1,2,3 \right)$ at age *t* by

$$h_{rr'}\left( t \right)=h_{rr'}^{0}\left( t \right)e^{\boldsymbol{\beta}_{rr'}^{'}\boldsymbol{X+}\gamma_{rr'}Z(t)},$$

where $h_{rr'}^{0}\left( t \right)=\lambda_{rr'}\tau_{rr'}t^{\tau_{rr'}-1}$ denotes the transition-specific Weibull-baseline hazard, $\boldsymbol{X}$denotes the vector of time-independent (baseline) covariates with corresponding parameter estimates $\boldsymbol{\beta}_{\boldsymbol{rr'}}$, and $Z(t)$ denotes the value of the pubertal effect function at age $t$with corresponding parameter estimates $\gamma_{rr'}$**.**

1. **Pubertal effect function**

We modelled the pubertal effect function as a function that could arise before the pubertal onset, reaches a steady state, and then vanishes. The function was of the form:

$$Z_{i}\left( t \right)=\left\{ \begin{aligned} \begin{aligned} \begin{aligned} \begin{matrix} 0, & when t<T_{0} \end{matrix} \\ \begin{matrix} \Delta^{-1}\left( t-T_{0} \right)_{+}, & when T_{0}\leq t\leq T_{1} \end{matrix} \\ \begin{matrix} 1, & when T_{1}<t\leq T_{2} \end{matrix} \end{aligned} \\ \begin{matrix} 1-\Delta^{-1}\left( t-T_{2} \right)_{+}, & when T_{2}<t\leq T_{3} \end{matrix} \end{aligned} \\ \begin{matrix} 0, & when t>T_{3} \end{matrix}, \end{aligned} \right.$$

where $t$ is the age, and the entire pubertal effect is assumed to last from $T_{0}$ to $T_{3}$, with the first (the last) $\Delta$ years representing a ramping up (fading off) period of the effect so that $\Delta=T_{1}-T_{0}=T_{3}-T_{2}$. During the steady period from $T_{1}$ to $T_{2}$, the pubertal effect is assumed to be steady. The estimated regression coefficient for $\gamma_{rr'}$ represents the maximal pubertal effect, which takes place during the steady period. During the ramping up and fading off periods, the current effect is obtained by multiplication of the parameter estimate with the current value of the pubertal effect function $Z(t)$. In our implementation, we used a discretization of time for the pubertal effect function by using 2-week time intervals.

1. **Simulations**
   1. **Setting**

We verified the appropriate performance of the model selection by simulations. Given survival up to time $u>0$, the cumulative distribution function $F(t|u)=1-e^{-\lambda\left( t^{\tau}-u^{\tau} \right)}$ can be used to simulate conditional Weibull event times by the inversion method. ^1^ We first simulated the ages at pubertal onset from $N(11.1, 1)$ based on the distribution of the actual ages at pubertal onset in the real data, and then times to islet autoimmunity (1 → 2) from $Weibull\left( {e^{\gamma_{12}Z(t)}\lambda}_{12},\tau_{12} \right)$ distribution, following by the times from islet autoimmunity to type 1 diabetes (2 → 3, progression) from $Weibull\left( {e^{\gamma_{23}Z(t)}\lambda}_{23},\tau_{23} \right)$ distribution, by conditioning the latter one on survival up to time of islet autoimmunity. During an individual pubertal period, repeated time draws were taken in a narrow time intervals of 2 weeks, by conditioning on survival up to beginning of the current interval (which is start of the pubertal period for the first interval). Draws were taken until the obtained time lied inside the particular 2-week time interval, or until the individual pubertal period ended. Finally, the data were adapted to include a 6-month follow-up for islet autoimmunity.

All the simulations were implemented with two different sample sizes: $n=1000$ and $n=5000$, both being less than the number of the individuals in the original dataset. Values for model parameters $\lambda_{12}=e^{-4.4}$, $\tau_{12}=e^{-0.5}$, $\gamma_{12}=0.3$, $\lambda_{23}=e^{-3}$, $\tau_{23}=e^{0.1}$, and $\gamma_{23}=0.45$ were chosen based on real data. We simulated 100 datasets and fitted four different models for all of them. Based on Akaike information criterion (AIC), we determined the model with the best fit for every dataset.

The data were simulated with the purpose to study to what extent the pubertal effect can be detected, and if so, whether the shape of the effect can be correctly identified. We ran four different models:

- **Model 1** included a pubertal effect with the origin at 1 year before the onset, a duration of 3 years, and a shape including 0.9-year (30%) ramping up, 1.2-year (40%) steady and 0.9-year (30%) fading off periods. This was the model the data were simulated from.
- **Model 2** had same origin and duration as model 1, but a different shape: of 0.3-year (10%) ramping up, 2.4-year (80%) steady and 0.3-year (10%) fading off periods (the pubertal effect used in the manuscript),
- **Model 3** had same origin and duration as in model 1, but a shape with only a steady (0%/100%/0%) period,
- **Model 4**: model without pubertal effect, i.e., $\gamma_{rr'} = 0$ for all *r, r’*.
  1. **Results**

The simulation with parameter choices leading to the relatively small number of events during a pubertal period, especially for islet autoimmunity (in real data with N=6920, n=15 for ICA+1-defined islet autoimmunity, and n=71 for ICA+1-defined progression) turned out to be challenging. With a smaller dataset ($n=1000$), the identification of any pubertal effect seemed to be difficult: a model with a pubertal effect was chosen only in 39% of the Monte-Carlo simulations (Supplementary Methods Table 1). The correct shape was detected in 17% of the simulations.

With a higher number of individuals ($n=5000$), the identification of the pubertal effect was substantially improved. It was detected among 78% of the simulations, and arrived to the correct shape of the effect in 36% of the runs (Supplementary Methods Table 1).

We compared the parameter estimates for pubertal effect obtained from the models from which data were simulated (Model 1), to the ones obtained from the models selected based on AIC. Despite of incorrectly chosen models, these estimates were rather similar with *n=5000* (Supplementary Methods Fig. 1). With *n=1000,* estimates of AIC-preferred models were slightly biased. This is likely due to large proportion of models with no pubertal effect being selected, also leading to small number of simulations (M=39) when those models suggesting the null were excluded from the presentation (Supplementary Methods Fig. 1). Overall, simulation results showed that at least with low number of events as in our data, sample size should be large to be able to detect the pubertal effect. For the correct identification of the shape of the effect, even more samples are needed. However, when pubertal effect is detected, the correct estimates for pubertal effect can be obtained despite of the unsuccessful shape identification.

**Supplementary Methods Table 1** The success of the data-driven model selection: numbers and percentages of the Akaike information criteria (AIC) based choices of the models with different pubertal function. Simulations are based on *M*=100 Monte-Carlo runs

| $M=100$ | Number of times chosen | |
| --- | --- | --- |
|  | n=1000 | n=5000 |
| Model 1 (30%/40%/30%) | 17 | 36 |
| Model 2 (10%/80%/10%) | 12 | 22 |
| Model 3 (0%/100%/0%) | 10 | 20 |
| Model 4 (No pubertal effect) | 61 | 22 |
| Total | 100 | 100 |


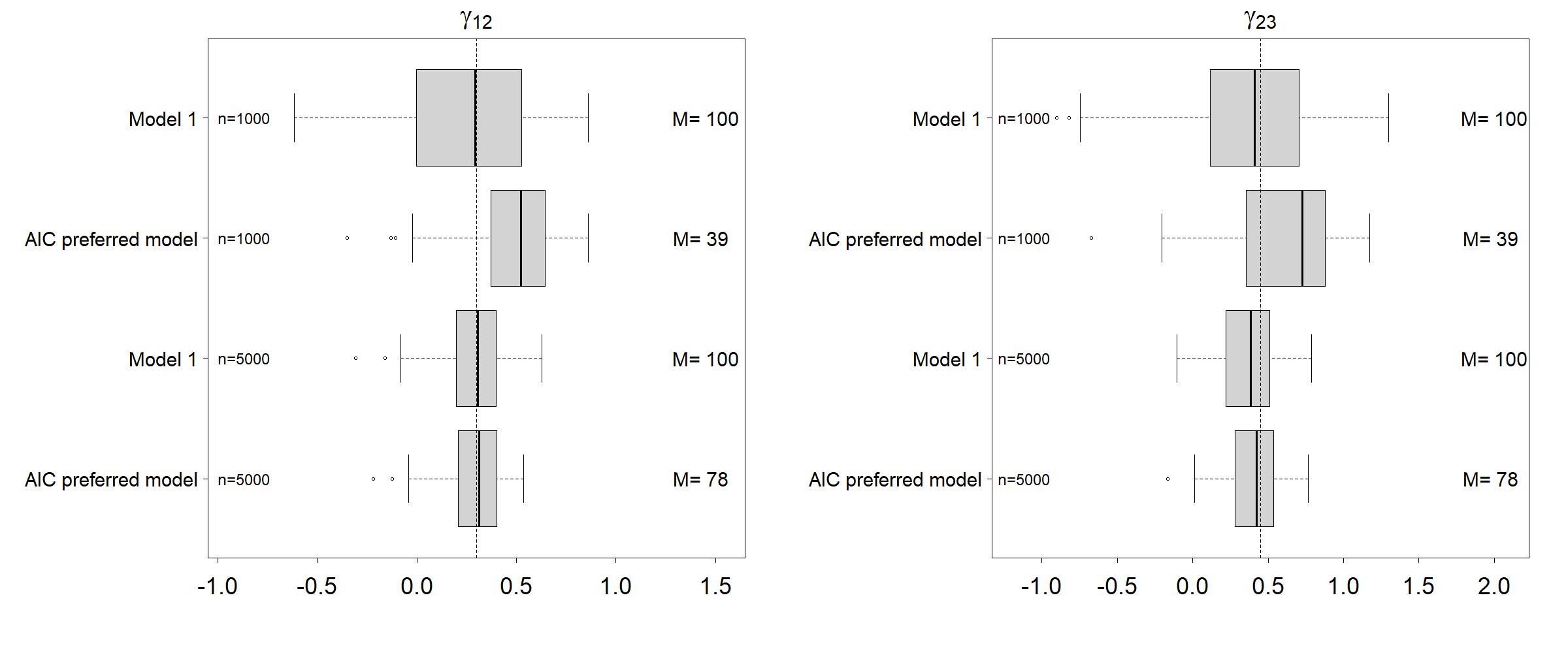


**Supplementary Methods Fig. 1** Distributions of the estimates for the pubertal effect from Model 1 (the model the data were simulated from), and the model which was selected as most suitable based on Akaike information criteria (AIC preferred model), separately for islet autoimmunity ($\gamma_{12}$) and progression from islet autoimmunity to type 1 diabetes ($\gamma_{23}$). The number of Monte-Carlo simulations was *M*=100. The estimates from the AIC preferred models wherein pubertal effect did not exist (Model 4) were not included leading to lower *M* for those models. Vertical dotted lines indicate the true parameter values

Supplementary References

1. Van Den Hout A. Multi-state survival models for interval-censored data: CRC Press; 2016.

stylefix
